# In Adult Rats With Polycystic Ovarian Syndrome, Unilateral or Bilateral Vagotomy Modifies the Noradrenergic Concentration in the Ovaries and the Celiac Superior Mesenteric Ganglia in Different Ways

**DOI:** 10.3389/fphys.2019.01309

**Published:** 2019-10-22

**Authors:** Rosa Linares, Gabriela Rosas, Elizabeth Vieyra, Deyra A. Ramírez, Daniel R. Velázquez, Julieta A. Espinoza, Carolina Morán, Roberto Domínguez, Leticia Morales-Ledesma

**Affiliations:** ^1^Laboratorio de Fisiología Reproductiva, de la Unidad de Investigación en Biología de la Reproducción, Facultad de Estudios Superiores Zaragoza, UNAM, Mexico City, Mexico; ^2^Centro de Investigación en Fisicoquímica de Materiales, Instituto de Ciencias, Benemérita Universidad Autónoma de Puebla, Puebla, Mexico

**Keywords:** PCOS, vagus nerve, vagotomy, hyperandrogenism, noradrenaline, celiac superior mesenteric ganglion

## Abstract

In rats with polycystic ovarian syndrome (PCOS) induced by estradiol valerate (EV) injection, sectioning of the vagus nerve in the juvenile stage restores ovulatory function, suggesting that the vagus nerve stimulates the onset and development of PCOS. We analyzed whether in adult rats, the role played by the vagus nerve in PCOS development is associated with the nerve’s regulation of noradrenergic activity in the celiac superior mesenteric ganglion (CSMG). Ten-day-old rats were injected with corn oil [vehicle (Vh)] or EV (2 mg). At 76 days of age, rats injected with Vh or EV were subjected to sham surgery or the sectioning of one or both vagus nerves (vagotomy). The animals were sacrificed at 80–82 days of age at vaginal estrus smear. Compared to Vh-treated animals, EV-induced PCOS rats showed a lack of ovulation, the presence of follicular cysts, and a high concentration of testosterone, without changes in noradrenaline concentrations in the CSMG or ovaries. In PCOS rats, sham surgery lowered serum testosterone and noradrenaline concentrations in the CSMG but did not restore ovulation. In animals with PCOS, vagotomy lowered testosterone concentrations to a larger degree than in sham-surgery animals. The ovaries of rats with PCOS and vagotomy showed fresh corpora lutea, indicating ovulation. In EV-treated rats with unilateral vagotomy, the concentration of noradrenaline in the CSMG was similar to that in rats with PCOS and sham surgery, which did not ovulate, while in the ovaries of PCOS rats with left or bilateral vagotomy, the noradrenaline concentration was lower than that in sham-surgery-treated animals. Our results suggest that the vagus nerve regulates PCOS development through a different mechanism than the increase in the noradrenergic activity in the CSMG; however, in ovaries, the restoration of ovulation is associated with a decrease in ovarian noradrenaline.

## Introduction

Evidence on the functional interaction between the peripheral nervous system and the reproductive system has been published (Domínguez and Riboni, 1971; Gerendai et al., 2000, 2009; Sosa et al., 2000; Casais et al., 2006). The ovary receives sympathetic innervation from the celiac superior mesenteric ganglia (CSMG) by two routes: the ovarian plexus nerve (OPN) and the superior ovarian nerve (SON) (Klein and Burden, 1988).

Experimental and clinical studies have shown the presence of multiple interactions between the sympathetic and parasympathetic nervous system, which are regulated through various pathways and mechanisms at the central and peripheral levels of the neuroaxis (Langer and Hicks, 1984; Myslivecek and Trojan, 2003). According to Ondicova and Mravec (2010), the peripheral interactions between the peripheral sympathetic and parasympathetic systems are based on the morphological-functional organization of the pathways. These interactions may be realized at the levels of the sympathetic prevertebral ganglia and neuroeffector connections. The CSMG is part of the sympathetic prevertebral chain and is the intermediate structure most closely related to the ovaries (Sosa et al., 2000, 2004; Morán et al., 2009). According to Fasano and Niel (2009) in the CSMG, there are three major types of cells, a set of neurons called principal ganglion cells, chromaffin cells, and glial cells, as well as a great variety of neurotransmitters with their respective receptors, such as noradrenaline and acetylcholine (Jan and Jan, 1983; Horn and Stofer, 1988). The CSMG also has a profuse capillary plexus forming circulatory microcircuits among the different ganglionic structures (Tanaka and Chiba, 1996).

According to Delgado et al. (2010), the vagus nerve is mainly composed of cholinergic fibers, most of which arrive at the CSMG. Additionally, Berthoud and Powley (1993, 1996) suggested that the interaction between the vagus nerve and the prevertebral ganglia may modulate the postganglionic outflow, allowing the vagal system to exert a more selective influence on sympathetic outflow.

Polycystic ovarian syndrome (PCOS) is characterized by a complex pathophysiology. An experimental model proposed to study PCOS is the administration of 2 mg of estradiol valerate (EV) to infantile or adult rats, which results in the development of a syndrome characterized by the interruption of the estrus cycle, persistent vaginal cornification, anovulation, the formation of follicular cysts, alterations to the basal and pulsatile concentrations of follicle-stimulating hormone (FSH) and luteinizing hormone (LH), as well as high concentrations of estradiol and testosterone (Barria et al., 1993; Rosa-E-Silva et al., 2003; Sotomayor-Zárate et al., 2008). These effects are similar to those observed in women with PCOS.

The primary etiology of PCOS remains unknown, though its association with increased sympathetic nerve activity has been identified (Barria et al., 1993; Lara et al., 1993; Sverrisdottir et al., 2008). In rats with PCOS induced by injecting EV, the expression of tyrosine hydroxylase in the CSMG (Lara et al., 1993) and the concentration of noradrenaline in the ovaries (Morales-Ledesma et al., 2010) are higher than in untreated animals. Both events were associated with the presence of precysts in the ovaries that precede the development of polycystic ovaries (Brawer et al., 1986; Lara et al., 2000, 2002; Rosa-E-Silva et al., 2003).

The participation of the vagus nerve in regulating ovarian functions has been suggested since the classic studies by Burden and Lawrence (1977, 1978), and Burden et al. (1983). We have previously shown that in 24-day-old rats with EV-induced PCOS, the unilateral or bilateral sectioning of the vagus nerve resulted in spontaneous ovulation in both ovaries (Linares et al., 2013) and decreased the noradrenaline concentration in the CSMG when the animal reached adulthood (Linares et al., 2017). These results suggest that the vagus nerve regulates the development of PCOS through its effects on the noradrenergic activity of CSMG.

To date, the main focus of these studies has been to analyze the role of the sympathetic hyperactivity reaching the ovaries via the SON, but the mechanisms regulating this hyperactivity are unknown. Thus, the present study aimed to analyze whether unilateral or bilateral vagotomy restores ovulation in the vaginal estrus cycle following surgery in adult rats and whether the role played by the vagus nerve in PCOS development is associated with the nerve’s regulation of noradrenergic activity in the CSMG.

## Materials and Methods

All experiments were carried out in strict accordance with the Mexican Law of Animal Treatment and Protection Guidelines. The committee of the Facultad de Estudios Superiores Zaragoza approved the experimental protocols. The study was performed using prepubertal female rats of the CIIZ-V strain from our breeding stock. Animals were kept under controlled lighting conditions (lights on from 05:00 to 19:00 h) with free access to rat chow pellets and tap water.

### Animal Treatment

Ten-day-old rats were injected with either a single dose of 0.1 ml of corn oil (vehicle (Vh)-treated *n* = 50) or 2 mg EV (Sigma Chem. Co., St. Louis, MO, United States) dissolved in 0.1 ml of corn oil (EV-treated *n* = 50). When Vh-treated and EV-treated rats reached 76 days of age, groups of ten animals were randomly assigned to one of the following groups:

(1) No surgery (Vh-control or EV-control).
(2) Sham-surgery (Vh-sham-surgery or EV-sham-surgery).
(3) Left vagus nerve sectioning (Vh-LVNS or EV-LVNS).
(4) Right vagus nerve sectioning (Vh-RVNS or EV-RVNS).
(5) Bilateral vagus nerve sectioning (Vh- BVNS or EV-BVNS).

Vagotomy and sham surgery procedures were performed between 10:00 and 12:00 h following previously described methodologies (Cruz et al., 1986; Linares et al., 2013, 2017). In brief, the rats were anesthetized with ether, and a ventral incision, including skin, muscle, and peritoneum, was made. Subsequently, the liver was retracted, the esophagus was exposed, and the left, right, or both vagal trunks were cut with fine forceps. Sham surgery involved the same procedures except that the vagus trunks were untouched. After surgery, the abdominal wall was sutured, and the animals returned to their cage.

### Autopsy Procedures

All animals were sacrificed at 80–82 days of age after vaginal smear indicated estrus preceded by diestrus or proestrus. All rats in the study were sacrificed by decapitation between 10:00 and 12:00 h. The blood from the trunk was collected, allowed to clot at room temperature, and centrifuged for 15 min at 3,000 rpm. The serum was stored at −20°C until progesterone, testosterone, and estradiol concentrations were measured. Following the criterion proposed by Burden and Lawrence (1977), observing a distended stomach during autopsy was considered a sign of functional vagotomy. The oviducts were dissected, and the number of oocytes ovulated was counted with the aid of a dissecting microscope. The location of the CSMG was identified according to the methodology described by Hammond and Kreulen (2016). In brief, the union between the celiac artery, the mesenteric artery and the aorta was located, and the adherent fatty tissue of the CSMG was removed and cleaned.

The ovaries and the CSMG of five animals were removed and stored at −70°C until monoamines were measured using high-performance liquid chromatography (HPLC). The rest of the ovaries were processed to perform the morphological analysis.

### Ovarian Morphology Assessment

The ovaries of Vh-control, EV-control and EV-treated rats with unilateral or bilateral vagotomy were cleaned of adherent fatty tissue, immersed in Bouin solution for 24 h, dehydrated, and embedded in paraffin. Ten-micron-thick serial sections were made and stained with hematoxylin-eosin. With the aid of a binocular microscope (Nikon model LabophoT-2, Japan), all ovarian sections were analyzed for the presence of fresh corpora lutea, healthy antral follicles and follicular cysts.

### Hormone Measurement

Progesterone (ng/ml), testosterone (pg/ml), and estradiol (pg/ml) concentrations in serum were measured using radioimmunoassay with kits purchased from Diagnostic Products (Los Angeles, CA, United States). The intra- and interassay coefficients of variation were 7.46 and 8.43% for progesterone, 8.75 and 9.48% for testosterone and 7.83 and 8.74% for estradiol, respectively.

### Noradrenaline Concentrations

The concentration of noradrenaline in the CSMG and ovaries was measured following previously described methodologies (Ayala et al., 1998). In brief, the ovaries and the CSMG were weighed on a precision balance, individually homogenized in 300 μl of 0.1 N perchloric acid, and subsequently centrifuged at 12,000 g for 30 min at −4°C. The supernatant was filtered using 0.2 μm regenerated cellulose filters. Twenty microliters of this extract was injected into a chromatography column via a Rheodyne injection valve. The HPLC system consisted of an isocratic pump (L-250 model; Perkin Elmer Co., Norwalk, CT, United States), a Rheodyne injection valve (7125 model; Perkin Elmer Co.), an Ultrasphere ODS preanalytical column (5 cm × 4.6 mm), and a Biophase ODS C-18 analytical column (25 cm 34.6 mm, 5 μm particle size; Bioanalytical Systems Inc., West Lafayette, IN, United States). The monoamine content in tissue was detected electrochemically using an LC-4A amperometric detector and an LC-5A glassy carbon transducer cell at an 850-mV potential. The mobile phase consisted of 0.1 M citrate buffer (Merck-México, SA.) at pH 3.0, with 175 mg of 1-octane-sulfonic acid (Sigma Chemical Co., St. Louis, MO, United States), filtered and degassed under vacuum. Immediately after degassing, 20 ml of acetonitrile and 21.5 ml of tetrahydrofuran for chromatography (Merck, Darmstadt, Germany) were added until a total volume of 500 ml was reached. The mobile phase was pumped at a flow rate of 1.2 ml/min. The system was calibrated by producing a 0.1 to 2 ng/ml standard range curve. Stock standards (Sigma Chemical Co., Louis, MO, United States) were prepared and diluted with 0.1 M perchloric acid on the same day as the experiment. Monoamine concentrations were identified by comparing the relative retention times in the samples with stock standards. Using a 1020 Perkin Elmer Nelson integrator, noradrenaline concentrations were determined by comparing the standard with the highest peak obtained from the samples. The results are expressed as picograms of neurotransmitter per milligrams wet tissue. The sensitivity for noradrenaline was 0.01 ng.

### Statistical Analysis

The results are expressed as the mean ± standard error of the mean (SEM). The number of oocytes ovulated by ovulating animals was analyzed using the Kruskal-Wallis test, followed by the Mann-Whitney *U*-test. The percentage of ovulating animals was analyzed using the chi-squared test. Progesterone, testosterone, and estradiol concentrations in serum and noradrenaline concentrations in the CSMG and ovaries were analyzed using a two-way analysis of variance (ANOVA) followed by Tukeyś multiple comparisons test using the Graph Pad Prism 6 Software. Differences between two groups were analyzed using Student’s *t*-test. A probability value less than 0.05 was considered statistically significant.

## Results

### Effects of Sham Surgery in Vh- or EV-Treated Rats

Regardless of whether they were subjected to sham surgery, the rats injected with EV did not present oocytes in the oviducts or corpora lutea in the ovaries (Table 1).

**TABLE 1:** Percentage of ovulating animals and mean ± SEM number of total oocytes ovulated; progesterone, testosterone, estradiol serum concentrations; and noradrenaline concentration in celiac superior mesenteric ganglia (CSMG) and ovaries of rats treated with vehicle (Vh) or estradiol valerate (EV) at day 10 of life, without surgery (control) or with sham surgery (sham) at day 76 of life.

**Group**	**Percent of ovulation**	**Oocytes ovulated**	**Steroid hormones**			**Noradrenaline**	
				
			**Progesterone**	**Testosterone**	**Estradiol**	**CSMG**	**Ovaries**
			**(ng/mL)**	**(pg/mL)**	**(pg/mL)**	**(pg/mg of ganglia)**	**(pg/mg of ovaries)**
Vh-control	10/10 = 100%	8.7 ± 0.9	10.0 ± 2.0	24.0 ± 6.0	38.0 ± 7.0	4080.2 ± 1091.3	1033.5 ± 237.4
Vh-Sham	8/10 = 80%	9.7 ± 1.5	6.5 ± 1.5	34.9 ± 12.9	83.8 ± 18.2	6406.4 ± 534.1	1963.1 ± 236.5^b^
EV-control	0/10 = 0% ^a^	0.0	13.0 ± 2.0	88.0 ± 11^a^	64.0 ± 13.0	6806.4 ± 312.6	1322.8 ± 166.4
EV-Sham	0/10 = 0%	0.0	4.1 ± 0.2^c^	57.4 ± 3.0^c^	70.3 ± 8.9	4364.7 ± 387.7^c^	2851.9 ± 20.4^c^

*Animals were sacrificed on day 80–82 of life. ^*a*^ vs. Vh-Control *p* < 0.0001; ^*b*^ vs. Vh-Control *p* < 0.05; ^*c*^ vs. EV-Control *p* < 0.05 Student’s “*t*” test.*

The progesterone or estradiol concentrations were not modified by EV treatment, while the serum testosterone concentration was higher than that in the Vh-control group. Sham surgery in EV-treated rats resulted in lower serum progesterone or testosterone concentrations than those found in the EV-control group (Table 1).

The concentration of noradrenaline in the CSMG of the rats injected with EV and subjected to sham surgery was lower than in the control group injected with EV. In the ovaries of the animals injected with Vh or EV and subjected to sham surgery, the concentration of noradrenaline was higher than that in the control groups (Table 1).

### Ovarian Morphology

**FIGURE 1:**
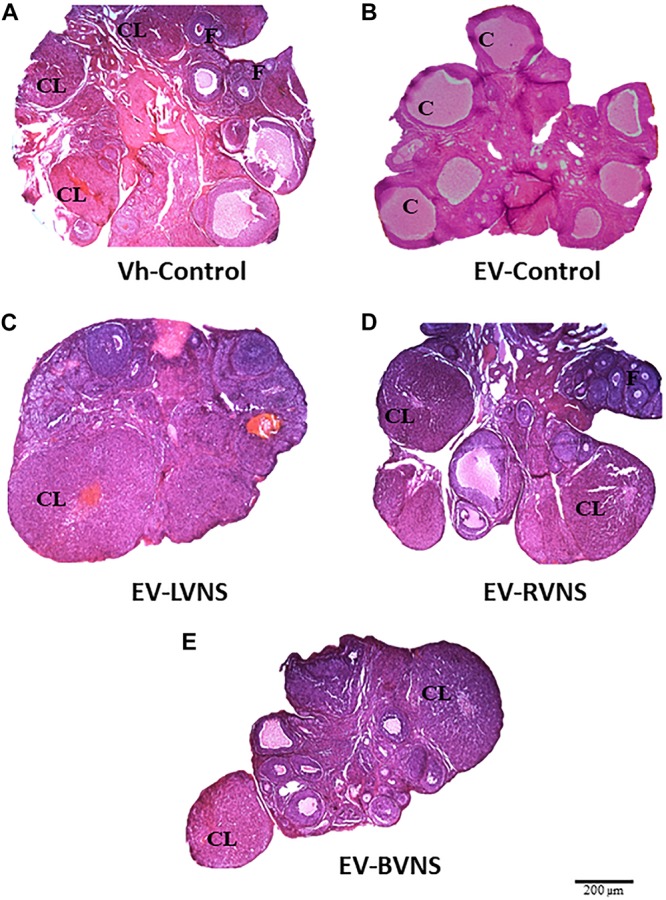
Ovarian histology in EV-induced PCOS rats after unilateral or bilateral vagotomy. Micrographs correspond to the largest section (10 μm thick) of the ovary from rats sacrificed the day of vaginal estrus (hematoxylin-eosin stain). **(A)** Ovary from a Vh-treated rat. **(B)** Polycystic ovary in an EV-treated rat. **(C–E)** Ovary from an EV-treated rat submitted to unilateral (LVNS or RVNS) or bilateral (BVNS) vagotomy at day 76 of life and sacrificed at day 80–82 of life. F, normal follicle; CL, corpora lutea; C, follicular cyst. 4 × microscopic lens. Scale bar = 200 μm.

Figure 1 shows the ovaries of a Vh-control animal sacrificed on estrus day containing corpora lutea and antral follicles (A). The ovaries of EV-control rats show cystic follicles and no corpora lutea (B), while the ovaries of EV-injected rats with unilateral or bilateral sectioning of the vagus nerve show fresh corpora lutea (C-E).

### Percentage of Ovulation and the Number of Oocytes Ovulated

No differences in the percentage of ovulating animals or the average number of oocytes released between right and left ovaries of rats injected with Vh or EV were observed. Therefore, the results obtained from each ovary were combined, and the results are reported as the percentage of ovulatory animals and the average number of oocytes released.

In the animals injected with Vh, right vagotomy resulted in a lower number of oocytes released than in the animals treated with Vh and sham surgery or in those treated with Vh and bilateral vagotomy. The rats injected with EV, subjected to sham surgery, did not present oocytes in the oviducts or corpora lutea in the ovaries. On the other hand, in the majority of the animals injected with EV and subjected to unilateral or bilateral vagotomy, oocytes in the oviducts and newly formed corpora lutea in the ovaries were found (Table 2).

**TABLE 2:** Percentage of ovulating animals and mean ± SEM number of total oocytes ovulated by rats treated with vehicle (Vh) or estradiol valerate (EV) at day 10 of life, with sham surgery (sham) or unilateral (LVNS or RVNS) or bilateral (BVNS) vagotomy at day 76 of life.

**Group**	**Percent of ovulation**	**Value of *p***	**Oocytes ovulated**	**Value of *p***
Vh-Sham	8/10 = 80%		9.7 ± 1.5	
EV-Sham	0/10 = 0%	*P* < 0.0007 vs. Vh-Sham		*P* < 0.0001 vs.Vh-Sham
Vh-LVNS	7/10 = 70%		7.0 ± 1.4	
EV-LVNS	8/10 = 80%	*P* < 0.0007 vs. EV-Sham	5.0 ± 1.0	*P* < 0.01 vs. EV-Sham
Vh-RVNS	9/10 = 90%		5.6 ± 1.0	*P* < 0.05 vs. Vh-Sham
EV-RVNS	7/10 = 70%	*P* < 0.003 vs. EV-Sham	7.4 ± 0.6	*P* < 0.0001 vs. EV-Sham
Vh-BVNS	7/10 = 70%		10.1 ± 1.5	*P* < 0.05 vs. Vh-RVNS
EV-BVNS	9/10 = 90%	*P* < 0.0001 vs. EV-Sham	6.3 ± 0.9	*P* < 0.05 vs. Vh-BVNS; *P* < 0.0001 vs. EV-Sham

*Animals were sacrificed on day 80–82 of life. Square test for ovulation percentage; Kruskal-Wallis test followed by Mann-Whitney *U*-test for the number of ovulated oocytes.*

### Progesterone, Testosterone, and Estradiol Concentrations

**FIGURE 2:**
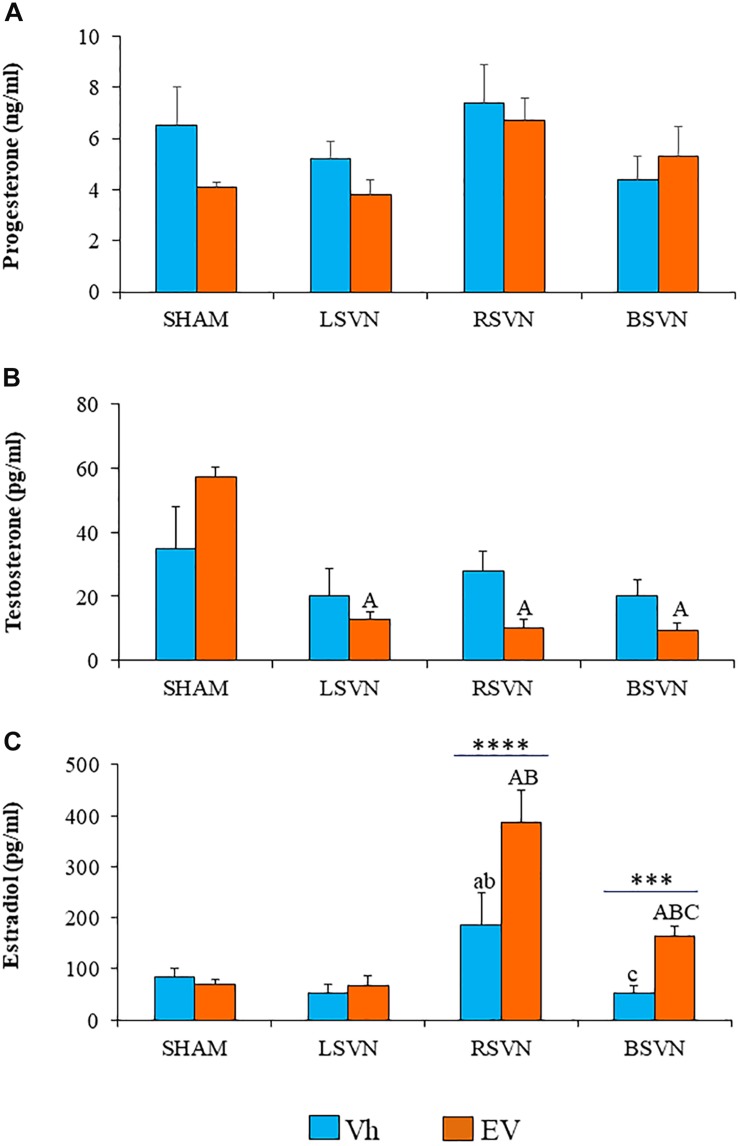
Steroid hormone concentrations. Mean ± SEM progesterone **(A)**, testosterone **(B)**, and estradiol **(C)** serum concentrations in rats injected with vehicle (Vh) or estradiol valerate (EV) at day 10 of life, with sham-surgery (sham) or unilateral (LVNS or RVNS) or bilateral (BVNS) vagotomy at day 76 of life, and sacrificed at day 80–82 of life. a vs. Vh-sham; b vs. Vh-LVNS; c vs. Vh-RVNS; A vs. EV-sham; B vs. EV-LVNS; C vs. EV-RVNS, two-way ANOVA followed by Tukeyś multiple comparisons test. The line indicates the difference between groups ^∗∗∗^*p* < 0.001, ^∗∗∗∗^*p* < 0.0001, Student’s *t*-test.

In Vh- and EV-treated rats, unilateral or bilateral vagotomy did not modify the serum progesterone concentration (Figure 2A).

Unilateral or bilateral vagotomy in EV-treated rats resulted in lower serum testosterone concentrations than in the group subjected to sham surgery (*p* < 0.0001) (Figure 2B).

The concentration of estradiol in rats injected with Vh subjected to right vagotomy was higher than in the group subjected to sham surgery or left vagotomy, while bilateral vagotomy resulted in a lower concentration with respect to right vagotomy. Right or bilateral vagotomy in rats injected with EV resulted in a higher serum estradiol concentration than that found in the group subjected to sham surgery (*p* < 0.0001). In EV-treated rats, right vagotomy resulted in higher serum estradiol concentrations than in groups with left or bilateral vagotomy. Estradiol concentrations in EV-treated rats with bilateral vagotomy were higher than those in the corresponding Vh-treated group (Figure 2C).

### Noradrenaline Concentrations in the Celiac Superior Mesenteric Ganglia (CSMG) and Ovaries

Since no differences were observed between the left or right ganglion and ovaries in all the experimental groups, the results are expressed as the total noradrenaline concentration in ganglion and ovaries.

**FIGURE 3:**
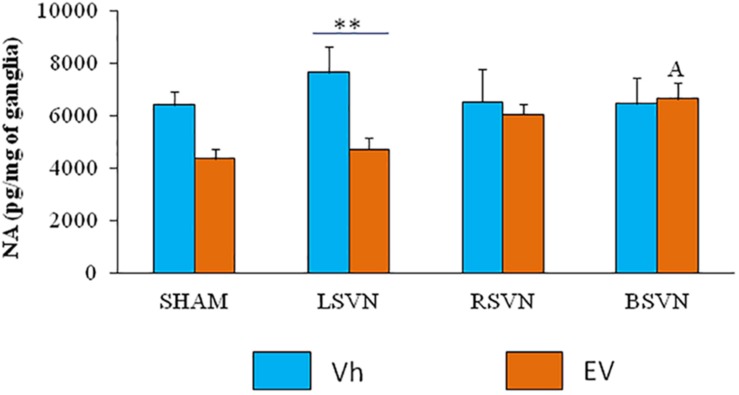
Noradrenaline concentration in the celiac superior mesenteric ganglia (CSMG). Mean ± SEM noradrenaline concentrations in the CSMG of rats treated with vehicle (Vh) or EV at day 10 of life, with sham-surgery (sham) or unilateral (LVNS or RVNS) or bilateral (BVNS) vagotomy at day 76 of life, and sacrificed at day 80–82 of life. A vs. EV-Sham, two-way ANOVA followed by Tukeyś multiple comparisons test. The line indicates the difference between groups ^∗∗^*p* < 0.01, Student’s *t*-test.

Left vagotomy in EV-treated rats resulted in a lower noradrenaline concentration than the same surgery in the Vh-treated group (*p* < 0.01). In contrast, in animals injected with EV, bilateral vagotomy resulted in a higher concentration of noradrenaline than sham surgery (Figure 3).

**FIGURE 4:**
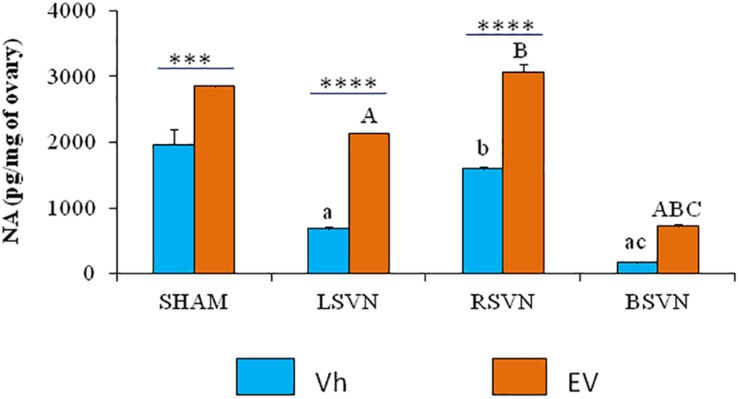
Noradrenaline concentration in the ovaries. Mean ± SEM noradrenaline concentrations in the ovaries of rats treated with vehicle (Vh) or EV at day 10 of life, with sham surgery (sham) or unilateral (LVNS or RVNS) or bilateral (BVNS) vagotomy at day 76 of life, and sacrificed at day 80–82 of life. a vs. Vh-sham; b vs. Vh-LVNS; c vs. Vh-RVNS; A vs. EV-sham; B vs. EV-LVNS; C vs. EV-RVNS, two-way ANOVA followed by Tukeyś multiple comparisons test. The line indicates the difference between groups ^∗∗∗^*p* < 0.001, ^∗∗∗∗^*p* < 0.0001, Student’s *t*-test.

The concentration of ovarian noradrenaline in animals injected with Vh subjected to left or bilateral vagotomy was lower than in the group with Vh-sham-surgery. The concentration of noradrenaline in the ovaries of the animals injected with Vh subjected to right vagotomy was higher than that in those subjected to left or bilateral vagotomy. In contrast, in the ovaries of the animals injected with EV subjected to left or bilateral vagotomy, the concentration of noradrenaline was lower than in the groups subjected to sham surgery or right vagotomy (Figure 4).

## Discussion

The present results suggest that in the adult rat, the development of PCOS is independent of the role that the vagus nerve has in the activity of the noradrenergic neurons of the CSMG.

According to Uchida et al. (2005), the acute unilateral stimulation of the abdominal wall of the female rat increased the ovarian sympathetic activity and the estradiol serum concentration during the time of stimulation, suggesting that nerve signals of peripheral origin participate in the mechanisms regulating estradiol secretion. In contrast, electrical stimulation of the SON reduces ovarian secretion of estradiol and blood flow, while the same stimulus on the ovarian plexus nerve reduces blood flow but does not modify estradiol secretion (Kagitani et al., 2008).

The density of sympathetic nerve fibers increases in the ovaries of rats treated with EV (Stener-Victorin et al., 2005), and according to Lara et al. (2000), the hyperactivation of the ovarian sympathetic input resulting from EV treatment is related to an overproduction of ovarian neural growth factor (NGF) and its low-affinity receptor in the ovary. In the present study, Vh-treated rats with sham surgery showed no differences in progesterone, testosterone or estradiol concentrations in serum or in noradrenaline concentrations in the CSMG. In these animals, the noradrenaline concentration in the ovaries was higher. In EV-treated animals with sham surgery, the ovarian steroid hormone concentrations in serum and noradrenaline concentrations in the CSMG were lower; however, the noradrenaline concentration in the ovaries was higher. We suggest that the different responses to sham surgery observed in Vh- and EV-injected rats are explained by changes in the innervation of the peritoneum and ovaries induced by EV treatment. Barco et al. (2003) proposed the existence of a neural pathway between the peritoneum and the ovaries and suggested that these neural pathways play significant roles in ovarian secretion.

According to Gerendai et al. (2000), there is a multisynaptic neural pathway between the ovary and several centers of the central nervous system (CNS), including vagal nuclei. The importance of the connection between the CNS and the ovaries has been evidenced by experiments in which the vagus nerve has been cut unilaterally (Morales et al., 2004; Linares et al., 2013) or bilaterally (Cruz et al., 1986; Morales et al., 2004). It has been suggested that the information that reaches the ovaries via the vagus nerve participates in the regulation of ovarian functions (Burden et al., 1983; Cruz et al., 1986; Trkulja and Lackovic, 2001; Morales et al., 2004, 2007).

Hemmings et al. (1983) showed that injecting luteinizing hormone releasing hormone (LHRH) into an EV-induced PCOS rat induces ovulation, suggesting that alterations in LHRH secretion by the hypothalamus are one of the main conditions that favor PCOS development and maintenance in the female reproductive system. On the other hand, we showed that in rats with EV-induced PCOS, the unilateral or bilateral vagotomy performed at 24 days of age resulted in ovulation in both ovaries 66–68 days after vagotomy treatment, without a change in gonadotropin concentration (Linares et al., 2013). In the present study, ovulation in both ovaries was confirmed 4–6 days after vagotomy treatment, suggesting that the effect of vagotomy on the reestablishment of ovulation is due to the immediate effect that the vagus nerve has on noradrenaline activity in the ovaries rather than the changes in gonadotropin concentration. In this regard, Allen et al. (1985) showed that vagotomy on the morning of proestrus did not prevent the proestrus LH surge and rats became estrus on the following day.

According to Tóth et al. (2007), the neural connections between the left ovary and brain structures are more abundant than those of the right gonad. Pastelín et al. (2017) showed that the neural pathways and sympathetic ganglia involved in the communication between the ovaries and the preganglionic neurons are different on the left and right side. The present results suggest that in rats with EV-induced PCOS, the vagus nerve plays an asymmetric role in regulating ovarian noradrenaline concentrations, since left vagotomy resulted in lower noradrenaline concentrations, while right vagotomy did not modify noradrenaline concentrations. We have previously shown that unilateral or bilateral sectioning of the SON in 24-day-old EV-injected rats resulted in decreased noradrenaline ovarian content (Morales-Ledesma et al., 2010). According to Lara et al. (2002), depending on the experimental animal model studied, the changes in noradrenaline concentration occur as a consequence of changes in sympathetic activity. Therefore, in the EV-injected rats, ovarian noradrenaline content seems to be regulated by neural information carried by the vagus nerve and the SON.

The cholinergic or noradrenergic stimulation of the CSMG modifies the release of ovarian steroids, providing physiological evidence of the participation of the sympathetic ganglionic pathway in the ovarian response (Sosa et al., 2000, 2004; Casais et al., 2006; Delgado et al., 2006; Vega-Orozco et al., 2006). According to Klein and Burden (1988), the fact that preganglionic fibers reaching the coeliac ganglia are of a cholinergic nature indicates that there exists a cholinergic modulation of the sympathetic postganglionic input to the ovary. Mravec (2011) proposed that vagal sensory fibers activated directly by adrenaline and noradrenaline represent the afferent extremity of a negative feedback loop that adjusts the activity of the sympatho-adrenal system according to the actual concentrations of plasma catecholamines and tissues. In the present study, bilateral vagotomy in rats with PCOS increased the noradrenaline concentration in the CSMG. It is possible that the vagus nerve also adjusts the activity of the sympatho-ovarian system.

The serum testosterone concentration in EV-induced rats with PCOS has been described as higher (Morales-Ledesma et al., 2010), lower (Rosa-E-Silva et al., 2003; Sotomayor-Zárate et al., 2008), and similar to those of control groups (Hemmings et al., 1983; Barria et al., 1993). In the present study, the testosterone concentration in EV-induced PCOS rats was higher than that in the Vh-treated group. According to Rosa-E-Silva et al. (2003), the rapid conversion of testosterone to estradiol in the ovary and/or its periphery can explain the lower testosterone concentrations they observed.

According to Stocco et al. (2007), in rodents, luteal cells continue to synthesize androstenedione and estradiol but become a site of substantial progesterone biosynthesis; the corpus luteum expresses high levels of key proteins involved in the uptake, synthesis, and transport of cholesterol and in the processing of cholesterol to progesterone and androgens as well as estrogens. Lawrence et al. (1978) showed that bilateral vagotomy on day 8 of pregnancy in rats decreased the activity of 3β-hydroxysteroid dehydrogenase in the corpus luteum and interstitial gland. Castro et al. (2001) showed that during the prepubertal development of the rat, a significant change in monoaminergic neural activity occurs within the anterior and medial hypothalamus and suggest that such a change may be linked to the development of neuroendocrine processes. We have previously shown that adult rats (90 days old) with bilateral vagotomy performed in the juvenile period (24 days old) present higher serum concentrations of progesterone, testosterone and estradiol than rats with sham surgery, while unilateral vagotomy did not modify hormone concentrations (Linares et al., 2013). The present results show that unilateral or bilateral vagotomy reversed the hyperandrogenism observed in rats with EV-induced PCOS and that right or bilateral vagotomy lowered estradiol levels. It is possible that the different effects of vagotomy performed in juvenile and adult rats on the concentration of steroid hormones are due to differences in the maturation of the vagal regulatory systems on steroid hormone secretion.

## Conclusion

In the adult rat, the persistence and development of PCOS are independent of the role that the vagus nerve has in the activity of noradrenergic neurons in the CSMG and apparently depend on the stimulating role of the nerve on the noradrenergic system of the ovary. The mechanism by which the vagus nerve modulates noradrenergic activity in the ovary is not yet clear. Based on the present and previous results, we suggest a combined involvement of the vagal and noradrenergic system that favors the formation of cysts.

## Data Availability Statement

All datasets generated for this study are included in the manuscript/supplementary files.

## Ethics Statement

All experiments were carried out in strict accordance with the Mexican Law of Animal Treatment and Protection Guidelines. The committee of the Facultad de Estudios Superiores Zaragoza approved the experimental protocols.

## Author Contributions

RL, LM-L, and RD planned the experiments. RL, GR, EV, DR, DV, JE, CM, RD, and LM-L devised the study and participated in the discussion of the results. RL and GR participated in performing the HPLC to measure the noradrenaline concentrations. All authors approved the final manuscript.

## Conflict of Interest

The authors declare that the research was conducted in the absence of any commercial or financial relationships that could be construed as a potential conflict of interest.

## Abbreviations

BVNS: bilateral vagus nerve sectioning
CSMG: celiac-superior mesenteric ganglion
EV: estradiol valerate
LVNS: left vagus nerve sectioning
PCOS: polycystic ovarian syndrome
RVNS: right vagus nerve sectioning
Vh: vehicle.

## References

[B1] Allen L. G. Lawrence I. E. Jr. Burden H. W. Hodson C. A. (1985). Effects of abdominal vagotomy on serum LH concentrations in female rats. *J. Reprod. Fertil.* 74 87–94. 10.1530/jrf.0.0740087 4040575

[B2] Ayala M. E. Monroy J. Morales L. Castro M. E. Domínguez R. (1998). Effects of a lesion in the dorsal raphe nuclei performed during the juvenile period of the female rat, on puberty. *Brain Res. Bull.* 47 211–218. 10.1016/s0361-9230(98)00074-4 9865852

[B3] Barco A. I. Flores A. Chavira R. Damián-Matsumura P. Domínguez R. Cruz M. E. (2003). Asymmetric effects of acute hemiovariectomy on steroid hormone secretion by the *In Situ* ovary. *Endocrine* 21 209–215. 14515003 10.1385/ENDO:21:3:209

[B4] Barria A. Leyton V. Ojeda S. Lara H. E. (1993). Ovarian steroidal response to gonadotropins and β-adrenergic stimulation is enhanced in polycystic ovary syndrome: role of sympathetic innervation. *Endocrinology* 133 2696–2703. 10.1210/endo.133.6.8243293 8243293

[B5] Berthoud H. R. Powley T. L. (1993). Characterization of vagal innervation to the rat celiac, suprarenal and mesenteric ganglia. *J. Auton. Nerv. Syst.* 42 153–169. 10.1016/0165-1838(93)90046-W 8450174

[B6] Berthoud H. R. Powley T. L. (1996). Interaction between parasympathetic and symphatetic nerves in prevertebral ganglia morphological evidence for vagal efferent innervation of ganglion cells in the rat. *Microsc. Res. Tech.* 35 80–86. 8873061 10.1002/(SICI)1097-0029(19960901)35:1<80::AID-JEMT7>3.0.CO;2-W

[B7] Brawer J. R. Munoz M. Farookhi R. (1986). Development of the polycystic ovarian condition (PCO) in the estradiol valerate-treated rat. *Biol. Reprod.* 35 647–655. 10.1095/biolreprod35.3.647 3098314

[B8] Burden H. W. Lawrence I. E. Jr. (1977). The effect of denervation on compensatory ovarian hypertrophy. *Neuroendocrinology* 23 368–378. 10.1159/000122685 563533

[B9] Burden H. W. Lawrence I. E. Jr. (1978). Experimental studies on the acetylcholinesterase-positive nerves in the ovary of the rat. *Anat. Rec.* 190 233–241. 10.1002/ar.1091900207 629404

[B10] Burden H. W. Leonard M. Smith C. P. Lawrence I. E. Jr. (1983). The sensory innervation of the ovary: a horseradish peroxidase study in the rat. *Anat. Rec.* 207 623–627. 10.1002/ar.1092070410 6670757

[B11] Casais M. Delgado S. M. Sosa Z. Y. Telleria C. M. Rastrilla A. M. (2006). The celiac ganglion modulates LH-induced inhibition of androstenedione release in late pregnant rat ovaries. *Reprod. Biol. Endocrinol.* 4 66–72. 10.1186/1477-7827-4-66 17184551 PMC1769501

[B12] Castro M. E. Ayala M. E. Monroy J. Chavira R. Damian-Matsumura P. Domínguez R. (2001). Changes in monoaminergic activity in the anterior, medium and posterior hypothalamus, gonadotropins levels and ovarian hormones during puberty of the female rat. *Brain Res. Bull.* 54 345–352. 10.1016/s0361-9230(00)00421-4 11306185

[B13] Cruz M. E. Chávez R. Domínguez R. (1986). Ovulation, follicular growth and ovarian reactivity to exogenous gonadotropins in adult rats with unilateral or bilateral section of the vagi nerves. *Rev. Invest. Clin.* 38 167–171. 3090668

[B14] Delgado S. M. Escudero C. G. Casais M. Anzulovich A. C. Sosa Z. Rastrilla A. M. (2010). Ovaric physiology in the first oestral cycle: influence of noradrenergic and cholinergic neural stimuli from coeliac ganglion. *Steroids* 75 685–694. 10.1016/j.steroids.2010.04.005 20433862

[B15] Delgado S. M. Sosa Z. Casais M. Rastrilla A. M. (2006). Ganglionic adrenergic action modulates ovarian steroids and nitric oxide in prepubertal rats. *Endocr. J.* 53 547–554. 10.1507/endocrj.K05-130 16849836

[B16] Domínguez R. Riboni L. (1971). Failure of ovulation in autografted ovary of hemispayed rat. *Neuroendocrinology* 7 164–170. 10.1159/000121964 5546027

[B17] Fasano C. Niel J. (2009). The mammalian sympathetic prevertebral ganglia: models for the study of neuronal networks and basic neuronal properties. *Auton. Neurosci.* 150 8–20. 10.1016/j.autneu.2009.06.006 19581130

[B18] Gerendai I. Tóth I. E. Boldogkoi Z. Halász B. (2009). Recent findings on the organization of central nervous system structures involved in the innervation of endocrine glands and other organs; observations obtained by the transneuronal viral double-labeling technique. *Endocrine* 36 179–188. 10.1007/s12020-009-9189-8 19418269

[B19] Gerendai I. Tóth I. E. Boldogkoi Z. Medveczky I. Halász B. (2000). CNS structures presumably involved in vagal control of ovarian function. *J. Auton. Nerv. Syst.* 80 40–45. 10.1016/s0165-1838(00)00071-0 10742538

[B20] Hammond B. Kreulen D. L. (2016). Gene therapy of the peripheral nervous system: celiac ganglia. *Methods Mol. Biol.* 1382 275–283. 10.1007/978-1-4939-3271-9_20 26611594

[B21] Hemmings R. Farookhi R. Brawer J. R. (1983). Pituitary and ovarian responses to luteinizing hormone releasing hormone in a rat with polycystic ovaries. *Biol. Reprod.* 29 239–248. 10.1095/biolreprod29.1.239 6351938

[B22] Horn J. P. Stofer W. D. (1988). Double labelling of the paravertebral sympathetic C system in the bullfrog with LHRH and NPY. *J. Auton. Nerv. Syst.* 23 17–25. 10.1016/0165-1838(88)90162-2 3049758

[B23] Jan Y. N. Jan L. Y. (1983). Coexistence and corelease of cholinergic and peptidergic transmitter in frog sympathetic ganglia. *Fed. Proc.* 12 2929–2935. 6136426

[B24] Kagitani F. Uchida S. Hotta H. (2008). Effects of electrical stimulation of the superior ovarian nerve and the ovarian plexus nerve on the ovarian estradiol secretion rate in rats. *J. Physiol. Sci.* 58 133–138. 10.2170/physiolsci.RP001508 18355419

[B25] Klein C. M. Burden H. W. (1988). Anatomical localization of afferent and postganglionic sympathetic neurons innervating the rat ovary. *Neurosc. Lett.* 85 217–222. 10.1016/0304-3940(88)90354-0 3374837

[B26] Langer S. Z. Hicks P. E. (1984). Physiology of the sympathetic nerve ending. *Br. J. Anaesth.* 56 689–700. 10.1093/bja/56.7.689 6145437

[B27] Lara H. Ferruz J. I. Luza S. Bustamante D. A. Borges Y. Ojeda S. R. (1993). Activation of ovarian sympathetic nerves in polycystic ovary syndrome. *Endocrinology* 133 2690–2695. 10.1210/endo.133.6.7902268 7902268

[B28] Lara H. E. Dissen G. A. Leyton V. Paredes A. Fuenzalida H. Fiedler J. L. (2000). An increased intraovarian synthesis of nerve growth factor and its low affinity receptor is a principal component of steroid induced polycystic ovary in the rat. *Endocrinology* 141 1059–1072. 10.1210/endo.141.3.7395 10698182

[B29] Lara H. E. Dorfman M. Venegas M. Luza S. M. Luna S. L. Mayerhofer A. (2002). Changes in sympathetic nerve activity of the mammalian ovary during a normal estrous cycle and in polycystic ovary syndrome: studies on norepinephrine release. *Microsc. Res. Tech.* 59 495–502. 10.1002/jemt.10229 12467025

[B30] Lawrence I. E. Jr. Burden H. W. Louis T. M. (1978). Effect of abdominal vagotomy of the pregnant rat on LH and progesterone concentrations and fetal resorption. *J. Reprod. Fertil.* 53 131–136. 10.1530/jrf.0.0530131 641892

[B31] Linares R. Hernández D. Morán C. Chavira R. Cárdenas M. Domínguez R. (2013). Unilateral or bilateral vagotomy induces ovulation in both ovaries of rats with polycystic ovarian syndrome. *Reprod. Biol. Endocrinol.* 1:68. 10.1186/1477-7827-11-68 23866168 PMC3722028

[B32] Linares R. Rosas G. Vieyra E. Ramírez D. A. Guerrero Y. A. Morán C. (2017). In rats with the polycystic ovary syndrome, the monoaminergic activity in the celiac superior mesenteric ganglion depends on the vagal innervation. *AMJ* 10 304–313. 10.21767/AMJ.2017.2878

[B33] Morales L. Betanzos R. Domínguez R. (2004). Unilateral or bilateral vagotomy performed on prepubertal rats at puberty onset of female rat deregulates ovarian function. *Arch. Med. Res.* 35 279–283. 10.1016/j.arcmed.2004.03.007 15325500

[B34] Morales L. Ricardo B. Bolaños A. Chavira R. Domínguez R. (2007). Ipsilateral vagotomy to unilaterally ovariectomized pre-pubertal rats modifies compensatory ovarian responses. *Reprod. Biol. Endocrinol.* 5:24. 10.1186/1477-7827-5-24 17567910 PMC1920514

[B35] Morales-Ledesma L. Linares R. Rosas G. Morán C. Chavira R. Cárdenas M. (2010). Unilateral sectioning of the superior ovarian nerve of rats with polycystic ovarian syndrome restores ovulation in the innervated ovary. *Reprod. Biol. Endocrinol.* 8:99. 10.1186/1477-7827-8-99 20723258 PMC2936316

[B36] Morán C. Zarate F. Morán J. L. Handal A. Domínguez R. (2009). Lateralization of the connections of the ovary to the celiac ganglia in juvenile rats. *Reprod. Biol. Endocrinol.* 7:50. 10.1186/1477-7827-7-50 19460167 PMC2697162

[B37] Mravec B. (2011). Role of catecholamine-induced activation of vagal afferent pathways in regulation of sympathoadrenal system activity: negative feedback loop of stress response. *Endocr. Regul.* 45 37–41. 21314209

[B38] Myslivecek J. Trojan S. (2003). Regulation of adrenoceptors and muscarinic receptors in the heart. *Gen. Physiol. Biophys.* 22 3–14. 12870697

[B39] Ondicova K. Mravec B. (2010). Multilevel interactions between the sympathetic and parasympathetic nervous systems: a minireview. *Endocr. Regul.* 44 69–75. 10.4149/endo_2010_02_69 20429636

[B40] Pastelín C. F. Rosas N. H. Morales-Ledesma L. Linares R. Domínguez R. Morán C. (2017). Anatomical organization and neural pathways of the ovarian plexus nerve in rats. *J. Ovarian Res.* 10:18. 10.1186/s13048-017-0311-x 28292315 PMC5351206

[B41] Rosa-E-Silva A. Guimaraes M. A. Padmanabhan V. Lara H. E. (2003). Prepubertal administration of estradiol valerate disrupts cyclicity and leads to cystic ovarian morphology during adult life in the rat: role of sympathetic innervation. *Endocrinology* 144 4289–4297. 10.1210/en.2003-0146 12960066

[B42] Sosa Z. Delgado S. M. Casais M. Aguado L. Rastrilla A. M. (2004). Release of ovarian progesterone during the rat oestrous cycle by ganglionic cholinergic influence: the role of norepinephrine. *J. Steroid Biochem. Mol. Biol.* 91 179–184. 10.1016/j.jsbmb.2004.03.119 15276625

[B43] Sosa Z. Y. Casais M. Rastrilla A. M. Aguado L. I. (2000). Adrenergic influences on coeliac ganglion affect the release of progesterone from cycling ovaries: characterization of an *in vitro* system. *J. Endocrinol.* 166 307–318. 10.1677/joe.0.1660307 10927620

[B44] Sotomayor-Zárate R. Dorfman M. Paredes A. Lara H. E. (2008). Neonatal exposure to estradiol valerate programs ovarian sympathetic innervation and follicular development in the adult rat. *Biol. Reprod.* 78 673–680. 10.1095/biolreprod.107.063974 18077802

[B45] Stener-Victorin E. Ploj K. Larsson B. M. Holmäng A. (2005). Rats with steroid- induced polycystic ovaries develop hypertension and increased sympathetic nervous system activity. *Reprod. Biol. Endocrinol.* 3:44. 10.1186/1477-7827-3-44 16146570 PMC1236959

[B46] Stocco C. Telleria C. Gibori G. (2007). The molecular control of corpus luteum formation, function, and regression. *Endocr. Rev.* 28 117–149. 10.1210/er.2006-0022 17077191

[B47] Sverrisdottir Y. B. Mogren T. Kataoka J. Janson P. O. Stener-Victorin E. (2008). Is polycystic ovary syndrome associated with high sympathetic nerve activity and size at birth? *Am. J. Physiol. Endocrinol. Metab.* 294 E576–E581. 10.1152/ajpendo.00725.2007 18198350

[B48] Tanaka K. Chiba T. (1996). Microvascular organization of sympathetic ganglia, with special reference to small intensely-fluorescent cells. *Microsc. Res. Tech.* 2 137–145. 8923448 10.1002/(SICI)1097-0029(19961001)35:2<137::AID-JEMT4>3.0.CO;2-N

[B49] Tóth I. E. Wiesel O. Boldogkoi Z. Bálint K. Tapaszti Z. Gerendai I. (2007). Predominance of supraspinal innervation of the left ovary. *Microsc. Res. Tech.* 70 710–718. 10.1002/jemt.20456 17393475

[B50] Trkulja V. Lackovic Z. (2001). Vagal influence on compensatory ovarian growth is important only briefly after hemicastration. *Exp. Biol. Med.* 226 776–781. 10.1177/153537020222600810 11520944

[B51] Uchida S. Kagitani F. Hotta H. Hanada T. Aikawa Y. (2005). Cutaneous mechanical stimulation regulates ovarian blood flow via activation of spinal and supraspinal reflex pathways in anesthetized rats. *Jpn. J. Physiol.* 55 265–277. 10.2170/jjphysiol.R2133 16259648

[B52] Vega-Orozco A. Sosa Z. Fillipa V. Mohamed F. Rastrilla A. M. (2006). The cholinergic influence on the mesenteric ganglion affects the liberation of ovarian steroids and nitric oxide in oestrus day rats: characterization of an *ex vivo* system. *J. Endocrinol.* 191 587–598. 10.1677/joe.1.06859 17170216

